# Glycemic variability correlates with medial temporal lobe atrophy and decreased cognitive performance in patients with memory deficits

**DOI:** 10.3389/fnagi.2023.1156908

**Published:** 2023-07-18

**Authors:** Shuangmei Zhang, Anrong Wang, Shen Liu, Hongyu Liu, Weifeng Zhu, Zhaoxu Zhang

**Affiliations:** ^1^Department of Pain Rehabilitation, Cancer Hospital of the University of Chinese Academy of Sciences, Zhejiang Cancer Hospital, Hangzhou, China; ^2^Institute of Cancer and Basic Medicine, Chinese Academy of Sciences, Hangzhou, China; ^3^The First Affiliated Hospital of Shandong First Medical University and Shandong Provincial Qianfoshan Hospital, Jinan, China; ^4^Department of Neurology of Traditional Chinese Medicine, Dongzhimen Hospital, Beijing University of Chinese Medicine, Beijing, China; ^5^Institute for Brain Disorders, Beijing University of Chinese Medicine, Beijing, China; ^6^Affiliated Hospital of Traditional Chinese Medicine of Guangzhou Medical University, Guangzhou, China; ^7^Department of Neurology, Peking University People’s Hospital, Beijing, China

**Keywords:** glycemic variability, blood glucose variability, MoCA, MMSE, medial temporal atrophy, MTA

## Abstract

**Background:**

In the past, researchers have observed a significant link between glycemia and dementia. Medial temporal atrophy (MTA) is regarded as a common marker of dementia. The correlation between glycemic variability and MTA is unclear, and it has not been determined whether glycemic variability can be utilized as a biomarker of MTA and cognitive performance.

**Methods:**

The patients in a memory clinic who underwent brain MRI scans and cognitive assessments within the first week of their hospital visit, were enrolled. All participants underwent three fasting blood glucose and one HBA1c assessments on three self-selected days within 1 week of their first visit. The variability independent of the mean (VIM) was employed. Validated visual scales were used to rate the MTA results. The mini-mental state examination (MMSE) and Montreal Cognitive Assessment (MoCA) scales were employed to assess the cognitive functions of the participants. Spearman’s correlation and regression models were used to examine the relationship between the MMSE and MoCA scales, and also determine the link between the MRI characteristics and cognitive status, where vascular risk factors, educational status, age, gender, and mean glucose parameters served as covariates.

**Results:**

Four hundred sixty-one subjects completed the MMSE scale, while 447 participants completed the MoCA scale. Data analysis revealed that 47.72% of the participants were men (220/461), and the median age of the patients was 69.87 ± 5.37 years. The findings of Spearman’s correlation analysis exhibited a strong negative relationship between the VIM and MMSE score (*r* = −0.729, *P* < 0.01), and the MoCA score (*r* = −0.710, *P* < 0.01). The VIM was regarded as an independent risk factor for determining cognitive impairment in both the MMSE and MoCA assessments. The results were unaffected by sensitivity analysis. In addition, a non-linear relationship was observed between the VIM and MTA scores.

**Conclusion:**

The variability in the blood glucose levels, which was presented as VIM, was related to the reduced cognitive function, which was reflected by MMSE and MoCA scales. The relationship between the VIM and the MTA score was non-linear. The VIM was positively related to the MTA score when the VIM was less than 2.42.

## Introduction

A few researchers have estimated that people aged 60 and above will outnumber adolescents and youth by 2050 (Straussner and Senreich, 2020). Dementia, an age-related disease, has emerged as a global health concern, and the lack of effective treatment options suggests that it will continue to be a public health priority indefinitely (Aguera-Ortiz et al., 2020). Alzheimer’s disease (AD) is the most prevalent type of dementia, which is pathologically manifested as neurodegeneration. The most common MRI indicator for neurodegeneration is medial temporal atrophy (MTA), which is closely associated with the diagnosis of AD (Scheltens and van de Pol, 2012; Woodworth et al., 2022).

Diabetes and dementia are closely related to each other. According to the findings presented by the systematic review and meta-analysis of 144 prospective studies, prediabetes, diabetes, and alterations in disease-related biochemical markers could be employed for predicting the increased risk of dementia or other types of cognitive impairment (Xue et al., 2019). For example, by increasing protein glycation and triggering advanced glycation end products (AGEs) formation, hyperglycemia may contribute to a link between diabetes and cognition (Chou et al., 2019). Blood glycemia, on the other hand, can exhibit considerable changes over time, spanning minutes, hours, days, and months. This glycemic variability has garnered a lot of attention over the last two decades (Yu et al., 2020).

*In vitro* and *in vivo* studies showed that increased glucose variability is related to an increased generation of reactive oxygen species (ROS), which exposes the vasculature to oxidative stress (Ceriello et al., 2008). As a result, the neurological system is susceptible to glucose variability (Bragd et al., 2008; Arnold et al., 2018). A few studies investigated the connection between individual glucose variability and cognitive impairment in type 2 diabetes patients (Kim et al., 2015). However, the relationship between glycemic variability and MTA remained unclear. It is unknown whether glycemic variability can serve as a biomarker of MTA and cognitive performance. To address this knowledge gap, this study recruited patients from a memory clinic, where a majority of the patients reported amnestic deficits and dementia or were at high risk of developing dementia. This cross-sectional study investigated whether glycemic variability was associated with a decline in cognition. Herein, the relationship between glycemic variability, cognitive decline, and MTA was also determined.

## Materials and methods

### Subjects

In this study, 604 patients with impaired cognitive function were initially retroactively enrolled in the memory clinic of Peking University People’s Hospital and Affiliated Hospital of Traditional Chinese Medicine of Guangzhou Medical University, between 1 January 2016 and 30 June 2020. Herein, 668 patients, who had undergone MRI scans and obtained valid MRI images within the first week of their initial memory clinic visit, were enrolled in this study. The below-mentioned exclusion criteria were employed in this study: (1) patients exhibiting cognitive dysfunction caused by a central nervous infection, brain tumor, brain trauma, or brain injuries due to chemotherapy or radiotherapy; (2) patients with impaired cognitive function caused by nutritional, metabolic, or infectious factors, like thyroid dysfunction, vitamin B deficiency, brain damage because of syphilis or alcohol exposure; (3) patients with severe psychiatric disease or severe psychiatric symptoms affecting cognitive assessment; (4) patients with a severe hemorrhagic stroke or large vessel infarctions.

This study eventually enrolled 461 patients. All participants underwent a clinical evaluation and neurological examination. The following details of the participants were also recorded: age, gender, education level, history of previous strokes, coronary heart disease, diabetes mellitus, hypertension, hyperlipidemia, and smoking. After overnight fasting, blood tests for fasting glucose (acquired on 3 random days within the week), total cholesterol, low-density lipoprotein cholesterol, high-density lipoprotein cholesterol, triglycerides, and uric acid were performed and documented. After examining the clinical information, MRI scans, cognitive function, and blood tests, we carried out the initial clinical diagnosis at the memory clinic.

The criteria outlined in the Diagnostic and Statistical Manual of Mental Disorders IV (DSMIV) (Gmitrowicz and Kucharska, 1994; Rabe-Jablonska and Bienkiewicz, 1994) were used to define dementia. The guidelines defined by the National Institute of Neurological and Communicative Disorders and Stroke and Alzheimer’s Disease and Related Disorders Association (NINCDS-ADRDA) were employed to diagnose probable AD (Mckhann et al., 1984). The National Institute of Neurological Disorders and Stroke (NINDS) and the Association Internationale pour la Recherche et l’Enseignement en Neurosciences (AIREN) criteria were referred to define probable vascular dementia (VaD) (Roman et al., 1993). The new MCI criteria established in 2003 were used to define mild cognitive impairment (MCI) (Winblad et al., 2004). The subjective cognitive decline initiative (SCD-I) criteria were followed to characterize subjective cognitive decline (SCD) (Molinuevo et al., 2017).

### Glucose variability indices and measurements

The glucose hexokinase technique (Hitachi Automatic Analyzer 7600, Hitachi Co., Tokyo, Japan) was used to test fasting blood sugar (FBS) levels. A high-performance liquid chromatography (HPLC) technique (Bio-Rad VARIANT II TURBO, Hercules, USA) was used to determine HBA1c levels in whole blood samples. Herein, the participants completed three fasting blood glucose assessments and one HBA1c assessment on three self-selected days within 1 week of their first hospital visit. Peripheral blood was collected from the participants between 7 and 9 a.m. after they had fasted overnight, and all measurements were completed before 11 a.m. that same day.

Intraindividual variability in blood glucose levels between visits was defined as glucose variability. For diabetic patients, visit-to-visit variability in glucose measurements has been utilized as a predictor of between-day variability (Lin et al., 2014; Cuevas et al., 2022). Regular examination at each visit was used to determine the standard deviations (SDs) between the FBS glucose levels. In this study, the SDs and coefficients of variation (CVs) for FBS glucose levels were used as indicators of glucose variability (Lim et al., 2018). The CV (%) was derived by expressing the standard deviations as a percentage of the mean: (CV% = SD/mean × 100). Subsequently, the variability independent of the mean (VIM) method was employed (Luo et al., 2020). VIM was determined as 100 × SD ÷ mean beta, wherein beta denoted the regression coefficient that was dependent on the natural logarithm of SD for the natural log of the mean (Kim et al., 2018; Xu et al., 2022).

### MRI imaging

The included participants underwent brain MRIs in the radiology department of Peking University People’s Hospital, with the help of a 3.0 T scanner (GE 750 or GE 750W, WI, USA). As mentioned above, these MRI scans were conducted seven days following the patient’s initial visit. The MRI scans used T1-weighted, T2-weighted, fluid-attenuated inversion recovery (FLAIR), diffusion-weighted imaging (DWI), and susceptibility-weighted imaging (SWI). A validated visual scale, ranging from 0 to 4, was used to assess MTA on the coronal T1 sequence. The patients were classified into 2 different groups depending on their MTA degree (level) (Scheltens and van de Pol, 2012; de Flores et al., 2020; Woodworth et al., 2022), i.e., none or mild atrophy (levels 0–1) and intermediate to severe atrophy (levels 2–4).

The scans were evaluated by two independent neuroradiologists who were blind to the other physician’s assessment of the images and clinical data of the patient. If there was a disagreement, a consultation was held to reach a consensus.

### Confounding factors

All the demographic variables were obtained from the patient data presented in the medical records system. Hypertension was defined as patient who was receiving antihypertensive drugs or showed a mean sitting systolic blood pressure value of > 140 mmHg or diastolic blood pressure of > 90 mmHg. Hyperlipidemia was defined as a condition where the patient exhibits a fasting total cholesterol level > 240 mg/dL or undergoes treatment using lipid-lowering drugs. A patient was considered a current smoker if they had smoked at least one cigarette per day over the past 6 months. On the other hand, patients who had quit smoking for <6 months were defined as non-smokers.

### Cognitive assessment outcomes

To minimize short-term variations in blood glucose concentrations, all neurocognitive tests were carried out between 10:00 and 11:00 a.m. The cognitive examinations were carried out by a professional neuropsychologist who was unaware of the patient’s clinical information. The patient was assessed during the first visit with the help of the Chinese versions of the mini-mental state examination (MMSE) scale (Folstein et al., 1975) and the Montreal Cognitive Assessment (MoCA) scale (Nasreddine et al., 2005). Patients showed cognitive impairment if their MMSE score was ≤ 26 or their MoCA score was <26 (furthermore, one point was added to the MoCA scale of the patients if they had < 2 years of education).

### Statistical analysis

SPSS 19.0 was used to analyze the data (IBM Corp., Armonk, USA). After determining the normality of the data, the median [interquartile range (IQR)] was utilized to present the data consisting of continuous variables. The variations observed between the two groups were assessed using the Kruskal–Wallis test. The chi-square test was employed to evaluate the variations in the baseline features for categorical variables, and an independent *t*-test was used for continuous variables. Furthermore, the relationships between MTA and glucose variability and between glucose variability and cognitive status (MMSE and MoCA scores) were determined using Spearman’s correlation analysis. The coefficient of variation was used as a categorical variable in the generalized linear and logistic regressions (grouped into tertiles). A few classical risk factors like gender, age, and education levels of patients, were adjusted as classical risk variables for cognitive impairment. A few other risk variables, like a history of diabetes, hyperlipidemia, hypertension, and cerebrovascular diseases, were adjusted in this study. Finally, two-sided *P*-values < 0.05 indicated the statistical significance level.

## Results

**FIGURE 1:**
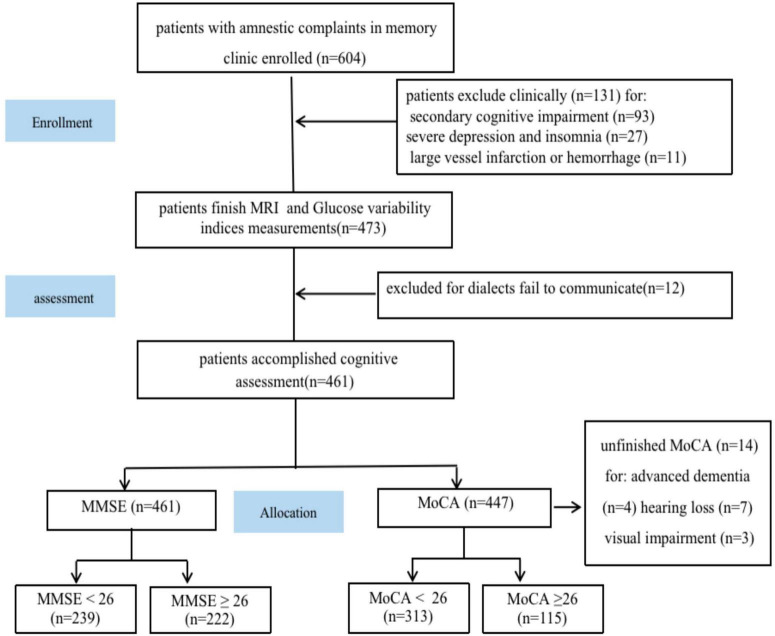
Study flowchart. MRI, magnetic resonance imaging; MoCA, Montreal Cognitive Assessment; MMSE, mini-mental state examination.

Out of the initial 704 patients who were enrolled in this study, 17 patients were removed due to brain injuries caused by traffic accidents, severe depression (17 patients), alcohol misuse (16 patients), severe carbon monoxide poisoning (16 patients), chemotherapy (15 patients), major artery infarctions, or severe cerebral hemorrhage (11 patients), encephalitis (11 patients), sleeplessness (10 patients), vitamin B deficiency (9 patients), and hypothyroidism (9 patients). Moreover, 12 patients were not included in this study because their cognition assessments were incomplete or invalid (which was attributed to poor communication owing to different language dialects). Additionally, 10 patients could not complete the MoCA due to severe hearing and visual impairments (because of cataracts and cancer). Four patients could not receive a valid MoCA diagnosis for advanced dementia, and their scales were (2, 3, 3, 4) (Figure 1). Thus, 461 patients completed the MMSE scale, and 447 completed the MoCA scale (Table 1).

**TABLE 1:** Baseline characteristics of study subjects.

Baseline characteristics	Total	Teriles of variability independent of the mean(VIM) in FBS			*P*
		Low	Middle	High	
**No. of participants**	461	154	152	155	
**Age (years)**	69.87 ± 5.37	69.25 ± 5.64	70.53 ± 5.33	69.85 ± 5.10	0.092
**Gender (male), n (%)**	220 (47.72%)	66 (42.86%)	71 (46.71%)	83 (53.55%)	0.163
**Years of education**	11.00 (8.00–13.00)	10.00 (8.00–12.00)	11.00 (9.00–12.00)	12.00 (11.00–15.00)	**<0.001**
**Duration, (years)**	2.00 (1.00–3.00)	2.00 (1.00–3.00)	2.00 (1.00–3.00)	2.00 (1.00–3.00)	0.154
**Hypertension, n (%)**	159 (34.49%)	33 (21.43%)	64 (42.11%)	62 (40.00%)	**<0.001**
**Diabetes, n (%)**	129 (27.98%)	48 (31.17%)	39 (25.66%)	42 (27.10%)	0.537
**Hyperlipidemia, n (%)**	66 (14.32%)	22 (14.29%)	19 (12.50%)	25 (16.13%)	0.662
**Heart disease, n (%)**	66 (14.32%)	24 (15.58%)	17 (11.18%)	25 (16.13%)	0.400
**Previous stroke, n (%)**	51 (11.06%)	15 (9.74%)	15 (9.87%)	21 (13.55%)	0.480
**Smoking, n (%)**	76 (16.49%)	24 (15.58%)	25 (16.45%)	27 (17.42%)	0.910
**Drinker, n (%)**	120 (26.03%)	28 (18.18%)	40 (26.32%)	52 (33.55%)	**0.009**
**Family history, n (%)**	38 (8.24%)	16 (10.39%)	12 (7.89%)	10 (6.45%)	0.445
**Living alone, %**	82 (17.79%)	22 (14.29%)	27 (17.76%)	33 (21.29%)	0.274
**HbA1c, %**	5.92 ± 1.09	5.94 ± 1.06	5.87 ± 1.03	5.96 ± 1.18	0.748
**FBS,mmol/l**	7.93 ± 1.06	7.58 ± 1.08	8.10 ± 1.11	8.11 ± 0.92	**<0.001**
**MTA**	1.00 (0.00–2.00)	1.00 (0.00–2.00)	2.00 (1.00–2.00)	2.00 (1.00–2.00)	**<0.001**
**MOCA**	21.44 ± 5.33	25.78 ± 3.04	21.19 ± 4.54	17.49 ± 4.56	**<0.001**
**MMSE**	23.22 ± 4.41	26.75 ± 2.19	23.17 ± 3.99	19.76 ± 3.62	**<0.001**

Results are presented as mean ± standard deviation or as a number (percentage) or median (Q1–Q3). FBS, fasting blood glucose; HBA1c, glycated hemoglobin; SD, standard deviation; IQR, interquartile range; MTA, medial temporal atrophy; bold values: P < 0.05.

According to the demographic data, 47.72% (220/461) of the patients were male, and the median age of all patients was recorded to be 69.87 ± 5.37 years. The median number of years of education was 11.0 years (8.0–13.0), and the median duration before their visit to the memory clinic was recorded to be 2.0 (1.0–3.0) years. The data revealed that 37.31% (172/461) of the patients were clinically diagnosed with dementia, 54.65% (94/172) of the dementia patients were diagnosed with probable AD, 11.05% (19/172) of the patients were diagnosed with probable VaD, and 34.30% (59/172) with other types of untyped dementia. It should be noted that 52.06% (240/461) of the patients had an MCI diagnosis, while 10.62% (49/461) of the patients were diagnosed with subjective cognitive impairment. The findings further revealed that 34.49% of the patients (159/461) had hypertension, 27.98% (129/461) had diabetes mellitus, and 14.32% (66/461) of the patients had hyperlipidemia coronary heart disease. It was also observed that 11.06% (51/461) of the patients had suffered from a previous stroke. Furthermore, the data revealed that 16.49% (76/461) of the patients were existing or former smokers, 26.03% (120/461) were alcohol consumers, and 8.24% (38/461) of the patients had a family history of dementia. All participants enrolled in this study underwent MMSE assessments and had a median score of 23.22 ± 4.41. Furthermore, 447 patients underwent MoCA assessment and had a median score of 21.44 ± 5.33 (Table 1).

No statistically significant variation was observed across different VIM groups in terms of age, gender, disease duration, hyperlipidemia, diabetes, previous stroke, heart disease, smoking, family history of dementia, living alone, or HBA1c. Patients in the other two groups displayed significantly lower MMSE and MoCA scores when compared to the lowest VIM group level (using tertiles) (Table 1).

### Relationship between VIM and cognitive status

According to the cognitive function scale assessment, 222 patients had an MMSE score of ≥26, while 135 patients had a MoCA score of ≥ 26. Thus, 239 patients displayed an MMSE score of < 26, and 313 patients showed a MoCA score of < 26, which indicated cognitive impairment. The data indicated that the patients with MMSE scores < 26 were older (69.08 ± 5.28 vs. 70.61 ± 5.37, *P* < 0.001), more educated (10.14 ± 2.86 vs. 11.93 ± 3.08, *P* < 0.001), had a longer duration before they visited the clinic [1.0 (1.0–3.0) vs. 2 (1.0–3.0), *P* < 0.001], had higher MTA levels [1.00 (0.00–2.00) vs. 2.00 (2.00–2.00), *P* < 0.001], and high MTA scores (1.58 ± 0.41 vs. 2.57 ± 0.60, *P* < 0.001). Additionally, the patients with MMSE scores < 26 consumed alcohol, suffered from hypertension, and lived alone more frequently compared to the patients with MMSE scores ≥ 26. No significant differences were observed between both groups based on their sex or other vascular risk factors (Table 2). Furthermore, a significant link was observed between the MMSE score and VIM levels (*r* = −0.729, *P* < 0.01) based on Spearman’s correlation analysis.

**TABLE 2:** Demographic, clinical characteristics of patients in different cognition status groups.

Baseline characteristics	MMSE ≥ 26 (N = 222)	MMSE < 26 (N = 239)	*P*	MOCA ≥ 26 (N = 135)	MOCA < 26 (N = 313)	*P*
Age (years)	69.08 ± 5.28	70.61 ± 5.37	0.003	68.16 (4.98)	70.55 (5.39)	<0.001
Gender (male), n (%)	106 (47.75%)	114 (47.70%)	0.992	62 (45.93%)	152 (48.56%)	0.608
Years of education	10.14 ± 2.86	11.93 ± 3.08	<0.001	10.02 (2.74)	11.50 (3.16)	<0.001
Duration, (years)	2.00 (1.00–3.00)	2.00 (1.00–3.00)	<0.001	2.00 (1.00–3.00)	2.00 (1.00–3.00)	0.078
Hypertension, n (%)	55 (24.77%)	104 (43.51%)	<0.001	30(22.22%)	127 (40.58%)	<0.001
Diabetes, n (%)	63 (28.38%)	66 (27.62%)	0.855	46 (34.07%)	80 (25.56%)	0.066
Hyperlipidemia, n (%)	34 (15.32%)	32 (13.39%)	0.555	20 (14.81%)	44 (14.06%)	0.834
Heart disease, n (%)	33 (14.86%)	33 (13.81%)	0.746	20 (14.81%)	43 (13.74%)	0.764
Previous stroke, n (%)	23 (10.36%)	28 (11.72%)	0.643	13 (9.63%)	36 (11.50%)	0.560
Smoking, n (%)	33 (14.86%)	43 (17.99%)	0.366	16 (11.85%)	55 (17.57%)	0.128
Drinker, n (%)	47 (21.17%)	73 (30.54%)	0.022	23 (17.04%)	91 (29.07%)	0.007
Family history, n (%)	24 (10.81%)	14 (5.86%)	0.053	14 (10.37%)	22 (7.03%)	0.233
Living alone, %	31 (13.96%)	51 (21.34%)	0.039	21 (15.56%)	58 (18.53%)	0.448
HbA1c, %	5.99 ± 1.08	5.86 ± 1.11	0.204	5.83 ± 1.06	5.96 ± 1.10	0.290
FBS,mmol/l	8.01 ± 1.18	7.86 ± 0.94	0.192	7.90 (1.21)	7.95 (0.99)	0.594
VIM	1.58 ± 0.41	2.57 ± 0.60	<0.001	1.46 (0.44)	2.37 (0.64)	<0.001
MTA	1.00 (0.00–2.00)	2.00 (2.00–2.00)	<0.001	1.00 (0.00–2.00)	2.00 (2.00–2.00)	<0.001

To evaluate the links between VIM and MMSE, a univariate linear regression model was used. Table 4 displays the adjusted and non-adjusted models. VIM showed a negative link to MMSE in the crude model [−4.26 (−4.67, −3.85), *P* < 0.001]. The findings of the minimally-adjusted model (adjusted for age and sex) and the fully adjusted model did not show any significant differences [−4.16 (−4.55, −3.76) *P* < 0.01 vs. −4.14 (−4.55, −3.74) *P* < 0.01]. In this study, VIM was used as a categorical variable (using tertiles) for the sensitivity analysis, and the findings showed a similar trend (*P* < 0.01). Thus, even after adjusting for gender, age, duration before visiting, education duration, diabetes, hyperlipidemia, hypertension, family history of dementia, smoking, living alone, consuming alcohol, HBA1c levels, FBS, and previous stroke, VIM remained an independent risk factor for cognitive impairment (MMSE < 26) (Table 4).

**TABLE 3:** Correlation analysis of VIM and MMSE, MoCA, MTA.

VIM	r	*P*
MMSE	−0.729	<0.01
MOCA	−0.710	<0.01
MTA	0.498	<0.01

**TABLE 4:** The correlation between VIM and MMSE, MoCA, MTA in different models.

Variable	Model 1		Model 2		Model 3	
MMSE	β (95%CI)	*P*	β (95%CI)	*P*	β (95%CI)	*P*
VIM	−4.26 (−4.67, −3.85)	<0.01	−4.16 (−4.55, −3.76)	<0.01	−4.14 (−4.55, −3.74)	<0.01
VIM tertile						
Low	Reference		Reference		Reference	
Middle	−3.58(−4.33, −2.82)	<0.01	−3.35 (−4.08, −2.63)	<0.01	−3.39 (−4.11, −2.67)	<0.01
High	−6.99(−7.73, −6.24)	<0.01	−6.921 (−7.64, −6.20)	<0.01	−6.85 (−7.59, −6.12)	<0.01
Pfor trend	−3.49 (−3.87, −3.12)	<0.01	−3.46 (−3.82, −3.10)	<0.01	−3.43(−3.80, −3.06)	<0.01
MOCA						
VIM	−4.95 (−5.46, −4.44)	<0.01	−4.82 (−5.32, −4.32)	<0.01	−4.614 (−5.13, −4.10)	<0.01
VIM tertile						
Low	Reference		Reference		Reference	
Middle	−4.59 (−5.53, −3.65)	<0.01	−4.33 (−5.23, −3.42)	<0.01	−4.12 (−5.04, −3.20)	<0.01
High	−8.28 (−9.22, −7.35)	<0.01	−8.19 (−9.09, −7.29)	<0.01	−7.74 (−8.68, −6.79)	<0.01
*P*for trend	−4.14(−4.60, −3.67)	<0.01	−4.09 (−4.54, −3.64)	<0.01	−3.86 (−4.33, −3.39)	<0.01
MTA						
VIM	0.70 (0.57, 0.82)	<0.01	0.69(0.57, 0.81)	<0.01	0.69 (0.55, 0.82)	<0.01
VIM tertile						
Low	Reference		Reference		Reference	
Middle	0.77 (0.55, 0.98)	<0.01	0.75 (0.54, 0.97)	<0.01	0.77 (0.55, 0.99)	<0.01
High	1.21 (0.99, 1.42)	<0.01	1.21 (0.99, 1.42)	<0.01	1.19 (0.96, 1.42)	<0.01
*P* for trend	0.60 (0.50, 0.71)	<0.01	0.60 (0.49, 0.71)	<0.01	0.59(0.48, 0.71)	<0.01

Model 1: adjust for: none; Model 2: adjust for: sex, age; Model 3: adjust for: sex, age, hypertension, hyperlipidemia, heart disease, previous stroke, smoking, family history, drinker, living alone, diabetes, HBA1c, FBS, education, duration.

Similar results were reported when the patients were assessed using the MoCA scale. Patients with a MoCA score < 26 [76.1% (223/293)] were observed to be older (68.16 ± 4.98 vs. 70.55 ± 5.39, *P* < 0.001), had more years of education (10.02 ± 2.74 vs. 11.50 ± 3.16, *P* < 0.001), as well as a higher percentage of hypertension [127 (40.58%)] and alcohol consumption [91 (29.07%)], compared to the patients with a MoCA score ≥ 26, who were less hypertensive [30 (22.22%)] and lower alcohol consumption [23 (17.04%)]. These patients also had a higher VIM level [1.46 (0.44) vs. 2.37 (0.64), *P* < 0.001]. Furthermore, both groups displayed insignificant differences based on the sex ratio or other vascular risk factors (Table 2). A significant relationship was observed between the VIM levels and MoCA score (*r* = −0.710, *P* < 0.01) (Table 3), based on Spearman’s correlation analysis. A univariate linear regression model was used for assessing the associations between VIM and MoCA. Table 4 presents the adjusted and non-adjusted models. VIM showed a negative link to MoCA in the crude model [−4.95 (−5.46, −4.44), *P* < 0.01]. The findings of the minimally-adjusted model (adjusted for sex and age) and the fully adjusted model did not show any significant differences [−4.82 (−5.32, −4.32) *P* < 0.01 vs. −4.614 (−5.13, −4.10) *P* < 0.01]. In this study, VIM served as a categorical variable (using tertiles) in sensitivity analysis, and the findings showed a similar trend (*P* < 0.01). VIM was an independent risk factor for cognitive impairment (MoCA < 26), after adjusting for gender, age, duration before visiting, years of education, diabetes, hyperlipidemia, hypertension, smoking, family history of dementia, alcohol intake, living alone, HBA1c, FBS, and the occurrence of a previous stroke (Table 4).

### Relationship between VIM and MTA

Medial temporal atrophy (MTA) is a common indicator of dementia (de Flores et al., 2020; Woodworth et al., 2022). Herein, the link between VIM, cognitive performance, and MTA was determined. Compared with the low VIM level group, patients presented a significantly high level of MTA (Table 1). Furthermore, in comparison with patients showing an MMSE score ≥ 26, patients with an MMSE score < 26 had higher MTA levels [1.00 (0.00–2.00) vs. 2.00 (2.00–2.00), *P* < 0.001] (Table 2). A significant link was observed between the MTA and VIM levels (*r* = 0.498, *P* < 0.01) based on Spearman’s correlation analysis (Table 3). A univariate linear regression model was employed for assessing the correlation between MTA and VIM. VIM exhibited a close correlation with MTA in the unadjusted, minimally adjusted, and fully adjusted models, and in the sensitivity analysis. Furthermore, a probable dose-response association was observed between the VIM tertiles and MTA levels. When compared to the low and middle VIM tertiles, the highest tertile participants displayed a significantly high MTA level [1.19 (0.96–1.42), *P* < 0.01] (the trend showed a *P*-value < 0.01). When all factors, including age, gender, years of education, time since the last visit, hypertension, hyperlipidemia, diabetes, smoking, drinking, living alone, FBS, HBA1c, and the incidence of a prior stroke, were adjusted, the findings remained significant.

**FIGURE 2:**
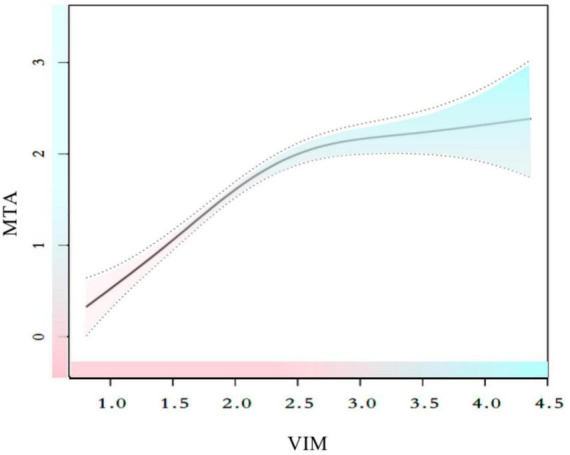
Smooth curve fitting for the relationships between VIM and MTA. The smooth curve fit between variables is represented by a solid line. The 95% confidence interval from the fit is represented by dotted line. Adjust for: sex, age, hypertension, hyperlipidemia, heart disease, previous stroke, smoking, family history, drinker, living alone, diabetes, HBA1c, FBS, education, duration.

Since VIM served as a continuous variable, the presence of non-linear correlations in the data was examined. After adjusting for gender, age, years of education, duration before visiting, family history of dementia, diabetes, hypertension, hyperlipidemia, smoking, alcohol consumption, living alone, FBS, HBA1c, and previous stroke, the connection between VIM and MTA was seen to be non-linear (Figure 2 and Table 5). The two-piecewise linear regression model was used to determine the inflection point as 2.42. The values of the effect size, 95% CI, and *P*-values at the left of the inflection point were 1.112 (0.887, 1.336) and *P* < 0.001, respectively. However, no relationship was observed between the VIM and MTA levels to the right of the inflection point [0.105 (−0.176, 0.387) *P* = 0.464] (Table 5).

**TABLE 5:** The results of two-piecewise linear regression model.

VIM	Adjusted β (95% CI), P
Fitting by a standard linear model	0.685 (0.553, 0.817) < 0.001
Fitting by two precise linear mode	
Inflection point	2.42
VIM < 2.42	1.112 (0.887, 1.336) < 0.001
VIM > 2.42	0.105 (−0.176, 0.387) 0.464
Log-likelihood ratio	<0.001

Effect: MTA. Cause: VIM. Sex, age, hypertension, hyperlipidemia, heart disease, previous stroke, smoking, family history, drinker, living alone, diabetes, HBA1c, FBS, education, duration were adjusted.

## Discussion

Cognitive decline occurs over a long period before the onset of dementia, and hence, it is essential to monitor individuals and provide early intervention to prevent or minimize cognitive deterioration (Nasreddine et al., 2005; Notarianni, 2022). Therefore, determining the risk factors for the cognitive decline may aid in identifying individuals who are at risk of dementia. It has been shown that hyperglycemia and diabetes are linked to cognitive decline and dementia progression (Ou et al., 2020; Papunen et al., 2020). The association of AGEs with cognitive changes in AD and T2DM patients was investigated in a high-quality clinical study conducted in Taiwan, and AGEs were associated with rapid declines in cognitive performance related to daily living among patients with AD and T2DM after 4 years (Chou et al., 2019). Many reports have determined the relationship between blood glucose levels and cognitive impairment, but a majority of the published studies have concentrated on diabetic individuals (Biessels et al., 2008; Shinohara and Sato, 2017; Sinclair and Abdelhafiz, 2020). A recent comprehensive review of the correlation between glucose regulation and cognition revealed that elevated HBA1c and glucose levels, as well as a higher level of glucose variability, are negatively correlated with cognitive function in elderly patients with type 2 diabetes (Philippou and Constantinou, 2014; Xue et al., 2019; Yu et al., 2020). Most of these studies determined that long-term hyperglycemia or hypoglycemic episodes affected the cognitive functions of non-diabetic or diabetic patients (Messier et al., 2011; Mehlig et al., 2018; Wang et al., 2019). However, very few reports studied the correlation between individual glucose variability and cognitive functions in the participants (Exalto et al., 2014; Burns et al., 2018). Additionally, this association has not been reported in patients with a primary complaint of amnesia. As a result, it is uncertain whether variation in glucose levels caused cognitive changes in amnesiac individuals. Hence, it is important to examine the correlation between glucose variability and cognitive impairment. For this purpose, the patients who were seeking treatment in a memory clinic with a main complaint of amnesia were investigated in this study.

Although blood glucose levels affect cognitive performance even in healthy individuals (Hawkins et al., 2016), amnesiac patients show a higher risk of dementia and cognitive decline compared to healthy people (Garcia-Casares et al., 2014; Geijselaers et al., 2015). Herein, the correlation between the VIM and MTA burden on the MRI results, and their effect on the cognitive performance of the memory clinic patients were examined. The MoCA and MMSE scales were used for assessing the overall cognitive functions, rather than assessing different cognitive categories because they were more practical during daily clinical screenings. The findings indicated that VIM was negatively related to the MMSE and MoCA scores. Spearman’s correlation analysis indicated a significant negative relationship between VIM and MMSE scores (*r* = −0.729, *P* < 0.001), and MoCA scores (*r* = −0.710, *P* < 0.001). Furthermore, regardless of whether the cognitive functions of the patients were evaluated using the MMSE or MoCA scales, VIM was used as an independent risk factor for cognitive impairment. The findings of a linear regression indicated that a high VIM could serve as a significant risk factor for the low MoCA score [β = −4.95, 95% CI (−5.46, −4.44), *P* < 0.01] and a low MMSE score [β = −4.26, 95% CI (−4.67, −3.85), *P* < 0.01]. This significance was still observed even after adjusting for factors such as education duration, gender, age, FBS, and conventional cardiovascular risk factors {the MMSE score [β = −4.14, 95% CI (−4.55, −3.74), *P* < 0.01] and the MoCA score [β = −4.614, 95% CI (−5.13, −4.10), *P* < 0.01]}. These findings were not significantly altered by sensitivity analysis (Table 4). The findings of this study indicate that some level of cognitive decline was related to elevated indices of glucose variability that were independent of mean glucose levels after completing the assessment, using either the MMSE or the MoCA cognitive tests, which were conducted prospectively by a neuropsychologist, who was unaware of patient history.

Visual assessment of MTA on MRI is frequently conducted in clinical settings, and MTA has been reported as an effective predictor of cognitive deterioration in a variety of patient cohorts, which includes participants in different stages of cognitive impairment (Jhoo et al., 2010; Garcia-Casares et al., 2014; Montandon et al., 2020), whether or not they are affected by cerebrovascular diseases. Earlier studies have demonstrated that high fasting glucose levels were strongly related to hippocampus and amygdala atrophy, which accounts for 6–10% of the volume changes after adjusting for different factors like age, gender, hypertension, body mass index, alcohol intake, and smoking (Ohara et al., 2020). Thus, this study investigated the correlation between blood glucose variability and the imaging results of MTA. MTA was also found to be a risk factor for cognitive dysfunction in this study, similar to the data presented in the earlier findings (Scheltens and van de Pol, 2012; Montandon et al., 2020). The results of Spearman’s correlation analysis showed a significant correlation between VIM and MTA levels (*r* = 0.498, *P* < 0.001), and linear regression analysis revealed that VIM could serve as a significant risk factor for MTA [β = 0.70, 95% CI (0.57, 0.82), *P* < 0.01]. Additionally, the sensitivity analysis (*P* for trend) and multiple imputations confirmed the stability of the relationship between VIM and MTA. However, a non-linear association was observed between VIM and MTA. A thorough literature review indicated that only a few studies examined the relationship between VIM and MTA. VIM and MTA correlated differently on the left and right sides of the inflection point (VIM = 2.42). VIM was significantly and positively linked to MTA on the left side of the inflection point [β = 1.112, 95% CI (0.887, 1.336), *P* < 0.01], but the relationship was insignificant on the right side of the inflection point [β = 0.105, 95% CI (−0.176, 0.387), *P* = 0.464].

A prior study showed that glycemic dysregulation can impair memory performance even at normal fasting glucose levels (Mortimer et al., 2010; Mortby et al., 2013). In this study, high glucose variability was inversely associated with memory patients’ cognitive test scores. This study displayed a significant relationship between blood glucose variability and low cognitive performance, which was similar to the findings presented in earlier studies (Kim et al., 2015; Lim et al., 2018). The present research identified an association between blood glucose variability and cognitive impairment, which could be attributed to MTA levels. A few earlier studies have reported that MTA levels were significantly and negatively correlated with cognitive performance (Jhoo et al., 2010; Montandon et al., 2020). However, earlier reports have also used a similar patient cohort as that used in this study.

Proper blood glucose control is critical to maintaining the brain health of diabetic patients (Gentreau et al., 2022). Glycemic variability negatively affects cognitive decline; however, the exact mechanism is unknown. The relationship between increased glucose variability, blood glucose levels, and brain health may be mediated by multiple potential processes. Firstly, it was hypothesized that the hippocampus was more vulnerable to hypoxia and glycemic control, in comparison to other regions of the brain (Cherbuin et al., 2012; Geijselaers et al., 2015; Gentreau et al., 2022). Secondly, different *in vitro* and *in vivo* studies showed that high glucose variability was related to increased ROS production, which exposes the vasculature to oxidative stress (Blazquez et al., 2022). The nervous system, and the medial temporal lobe, in particular, are vulnerable to glucose variability (Dewanjee et al., 2022). Thirdly, hyperglycemia-induced insulin secretion may lead to peripheral or cerebral insulin resistance, which is related to neurodegeneration, neuronal vulnerability, and other pathological lesions (Watt et al., 2020; Blazquez et al., 2022). Insulin receptor and signaling dysfunction in the brain may affect neuronal survival, nitric oxide-mediated vasodilation, astrocyte inflammatory cytokine secretion, and cerebral perfusion (Arnold et al., 2018). The medial temporal lobe is especially prone to ischemia and hypoperfusion (Burns et al., 2018). Fourth, protein cross-linking mediated by AGEs can promote amyloidosis in AD by accelerating polymerization of amyloid (Sun et al., 2020). It is also possible for AGEs to induce tau-protein hyperphosphorylation, stabilize the paired helical filament tau, and cause neurofibrillary tangles (Li et al., 2012; Lee et al., 2022). Finally, elevated blood fasting glucose levels have been significantly related to depression (Gilsanz et al., 2018), which is considered a risk factor for dementia.

Despite the above-mentioned advantages, this study had some limitations. Firstly, this was a cross-sectional, retrospective study, as it tested the associations but not causation for a specific population of patients in a memory clinic associated with two medical centers. This might limit the ability to extend the conclusions of this study to the broader population. Secondly, no follow-up was carried out for the patients included in this study, which affected the ability of this study to assess changes in cognitive impairment longitudinally. Finally, although this study highlighted the relationship between cognitive function performance, glucose variability, and MTA, this study was observational in nature and could not prove causation.

Some of the advantages of this study included a large number of subjects with both normal and abnormal brain MRI findings and the use of standardized diagnostic assessments. Subjectivity in the visual rating of the MRIs may have limited the scope of this study, which could lead to the overlooking of some important data. However, this method was less time-consuming and did not necessitate the use of any particular software. Even though this technique showed a small probability of some information being lost, it is still a useful tool for determining the MTA load in clinical practice and research. In this study, the popular MMSE and MoCA scales were selected as they focused on the overall cognitive functions instead of a definitive diagnosis, which was more suited for daily clinical practice and research. The linear relationships between VIM and MMSE, MoCA, and MTA were assessed using a generalized linear model, and the non-linear interactions were elucidated using a generalized additive model (GAM). The use of a GAM aided in determining the true correlations between exposure and outcome. The findings in this study demonstrated the negative effects of VIM and emphasized the need for maintaining appropriate glycemic control. To the best of our knowledge, this is probably the first study that examined the correlation between VIM and cognitive decline in patients with the primary complaint of amnesia, as well as the relationship between VIM and MTA. These results suggested that monitoring and control of plasma glucose levels could have a significant impact on cerebral health, even in the case of patients in the subclinical range and non-diabetic individuals.

## Conclusion

The findings of the MoCA and MMSE scales highlighted the correlation between impaired cognitive function and blood glucose variability measured by VIM. VIM and MTA displayed a non-linear relationship. It was seen that when VIM < 2.42, it was positively linked to MTA. These findings support the hypothesis that the VIM value and MTA score could be used to assess cognitive impairment. There is a need to carry out thorough, randomized, controlled trials to compare the effects of intensive glucose control and conventional glucose control strategies on brain structural abnormalities and dementia risk.

## Data availability statement

The raw data supporting the conclusions of this article will be made available by the authors, without undue reservation.

## Ethics statement

The studies involving human participants were reviewed and approved by the Ethics Committee of Peking University People’s Hospital and the Ethics Committee of Hospital of Traditional Chinese Medicine Affiliated to Guangzhou Medical University. The patients/participants provided their written informed consent to participate in this study.

## Author contributions

SZ and ZZ collected, analyzed, interpreted the patient data, designed this study, and was a major contributor in writing the manuscript. AW and SL made contributions to the acquisition of data. SZ and HL helped in the statistical analysis of the data. ZZ revised the manuscript and helped to interpret the data. All authors have read and approved the final version of this manuscript.
